# 有临床意义的单克隆免疫球蛋白血症的诊断及鉴别诊断中国专家共识（2022年版）

**DOI:** 10.3760/cma.j.issn.0253-2727.2022.08.003

**Published:** 2022-08

**Authors:** 中华医学会血液学分会 中国抗癌协会血液肿瘤专业委员会 中国少见浆细胞病协作组

有临床意义的单克隆免疫球蛋白血症（monoclonal gammopathies of clinical significance, MGCS）是一组以血或尿中存在单克隆免疫球蛋白（M蛋白）及其所造成的器官损害为主要临床特征的疾病。MGCS的疾病谱非常广泛、疾病表现多样，临床表现高度异质性，而且大多为少见/罕见疾病，因此容易被漏诊和误诊。为提高中国医师对这类疾病的认识及诊断水平，中华医学会血液学分会、中国抗癌协会血液肿瘤专业委员会和中国少见浆细胞病协作组特组织相关专家经过多次讨论，制定了本版MGCS的诊断与鉴别诊断专家共识。

一、概述

1. 定义：确诊MGCS通常需要满足以下条件：①血或尿中存在M蛋白；②M蛋白造成了直接或间接的器官损害；③排除多发性骨髓瘤、华氏巨球蛋白血症和B细胞淋巴瘤等肿瘤性疾病。

2. 疾病分类：

按照M蛋白类型可以将MGCS分成非IgM型MGCS和IgM型MGCS两大类，两种类型包含不同的疾病谱。非IgM型MGCS主要包括原发性轻链型淀粉样变、POEMS综合征、轻链沉积病、轻链近端肾小管病、硬化性黏液水肿、Clarkson病、散发迟发性杆状体肌病（SLONM）和TEMPI综合征等。而IgM型MGCS则主要包括原发性冷凝集素病（CAD）、冷球蛋白血症、IgM相关性周围神经病、CANOMAD综合征和Schnitzler综合征等。

按照疾病的生物学行为及M蛋白在疾病发生发展中的作用，可将MGCS分成以下几类：①以M蛋白或其组分沉积为主要表现的MGCS：如原发性轻链型淀粉样变、轻链沉积病和轻链近端肾小管病等；②以M蛋白相关的自身抗体效应为主要表现的MGCS：CAD、冷球蛋白血症和IgM相关性周围神经病等；③M蛋白在疾病发生中的作用尚不清楚的MGCS：POEMS综合征、硬化性黏液水肿、Clarkson病、SLONM和TEMPI综合征等。

二、M蛋白的检测方法

血清蛋白电泳（SPEP）、血/尿免疫固定电泳（IFE）及血清游离轻链（FLC）比值、重轻链（HLC）比值都可用于检测患者是否存在M蛋白。每种方法各有优缺点，在临床应用中应联合上述多种方法进行M蛋白的定性及定量检测。

1. SPEP：SPEP检测较为简便和普遍，技术要求较低。其不仅可以发现血中异常M蛋白，还可以对M蛋白进行定量。但是，SPEP的敏感性低，检测下限为0.3～0.5 g/L；且SPEP不能进行M蛋白分型。

2. 血/尿IFE：通过血/尿IFE检测可以对M蛋白进行具体分型。IFE的检测敏感度高于SPEP。血IFE可检出血中高于0.2 g/L的M蛋白，而尿IFE可检出尿中低至0.04 g/L的M蛋白。IFE不能定量检测。对于单纯轻链型患者，应加做IgD免疫固定电泳鉴定是否为IgD型M蛋白。

3. FLC检测：一般采用免疫散射比浊法检测血清FLC。通过可特异性结合FLC的针对免疫球蛋白轻链结合面表位的抗体，可进行极低浓度的FLC检测；并通过计算游离κ和游离λ的比值判读出轻链的克隆性。血清游离κ/λ比值的正常值为0.26～1.65，超过1.65代表存在单克隆κ型轻链；低于0.26则代表存在单克隆λ型轻链。其检测灵敏度（10～30 mg/L）较SPEP和IFE进一步提高，可以对微量M蛋白进行定量监测[1]。

4. 血清HLC检测：是近年来发展的利用免疫法特异性定量检测血清中同型特异性重/轻链对（如IgGκ和IgGλ），并利用HLC比值判断克隆性的方法[2]。例如，IgGκ/IgGλ正常值为1.3～3.7，IgAκ/IgAλ正常值为0.7～2.2，IgMκ/IgMλ正常值为1.0～2.4，超出上述范围意味着存在单克隆M蛋白。其临床应用尚在探索中，有条件的单位可以开展。

三、MGCS的诊断和鉴别诊断

1. 原发性轻链型淀粉样变

诊断：需满足以下5条标准：①有典型的器官受累的临床表现和体征；②血尿中存在单克隆免疫球蛋白；③组织活检可见无定形粉染物质沉积，刚果红染色阳性；④沉积物鉴定为免疫球蛋白轻链沉积；⑤除外症状性多发性骨髓瘤、华氏巨球蛋白血症或其他B细胞淋巴肿瘤。

危险分层：建议采用梅奥诊所的临床分期（2004分期或2012分期）。梅奥2004分期包括：Ⅰ期，肌钙蛋白T<0.035 µg/L或肌钙蛋白I<0.1 µg/L及NT-proBNP<332 ng/L；Ⅱ期，肌钙蛋白T≥0.035 µg/L或肌钙蛋白I≥0.1 µg/L或NT-proBNP≥332 ng/L；Ⅲ期，肌钙蛋白T≥0.035 µg/L或肌钙蛋白I≥0.1 µg/L及NT-proBNP≥332 ng/L[3]。可以按照NT-proBNP是否≥8 500 ng/L，将Ⅲ期患者进一步分为Ⅲa期和Ⅲb期[4]。梅奥2012分期包括3个危险因素：肌钙蛋白T≥0.025 µg/L；NT-proBNP≥1 800 ng/L；FLC差值（dFLC）≥180 mg/L。按照危险因素数目对患者分期：1期：0个危险因素；2期：1个危险因素；3期：2个危险因素；4期：3个危险因素[5]。

治疗目标及原则：轻链型淀粉样变的治疗目标是高质量的血液学缓解，即达到非常好的部分缓解（VGPR）及以上的血液学缓解。器官缓解往往发生在获得血液学缓解的6～12个月后。轻链型淀粉样变现有的核心治疗是抗浆细胞治疗，包括达雷妥尤单抗联合化疗、硼替佐米/环磷酰胺/地塞米松、自体造血干细胞移植或者美法仑联合地塞米松等[6]–[8]。

2. POEMS综合征

诊断：强制性诊断标准：①多发周围神经病；②单克隆浆细胞异常增殖（几乎均为λ轻链）。主要诊断标准：①Castleman病；②硬化性骨病；③血清或血浆血管内皮生长因子（VEGF）水平升高。次要诊断标准：①脏器肿大（肝大、脾大或淋巴结大）；②血管外容量过多（外周水肿、腹腔积液或胸腔积液）；③内分泌改变（肾上腺、甲状腺、垂体功能、性腺、甲状旁腺、胰腺）（单纯糖尿病或甲状腺功能异常不能作为诊断标准）；④皮肤病变（皮肤变黑、多毛、肾小球样血管瘤、多血质、发绀和白甲）；⑤视乳头水肿；⑥血小板增多或红细胞增多。诊断需同时满足两条强制性诊断标准、1条主要诊断标准和1条次要诊断标准[9]。

预后和治疗：POEMS综合征为慢性病程，其预后明显优于多发性骨髓瘤和原发性轻链型淀粉样变，10年生存率约为75％[10]。治疗主要为抗浆细胞治疗，如来那度胺联合地塞米松、硼替佐米联合地塞米松、美法仑联合地塞米松和自体造血干细胞移植[11]–[13]。

3. 轻链沉积病

诊断：①临床表现为无症状性镜下血尿、蛋白尿、高血压和（或）肾功能不全等。②肾活检病理是诊断轻链沉积病的金标准：光镜下可见结节硬化性肾小球肾炎、肾小管萎缩和间质纤维化伴无定形物质沉积；刚果红染色阴性；免疫荧光可见单一轻链沿基底膜线状沉积；电镜可见特征性颗粒状电子致密物沿基底膜下沉积。③血尿中存在单克隆免疫球蛋白，大部分为κ型轻链。肾脏受累最为常见，其他器官如心脏、肝脏、肺脏等亦可受累[14]–[15]。

治疗和预后：治疗原则与治疗目标均遵从轻链型淀粉样变的治疗。

4. 轻链近端肾小管病：是轻链（主要为κ型轻链）沉积在近端肾小管造成获得性近端肾小管功能障碍，临床上可表现为Fanconi综合征。

诊断：①主要临床表现：尿糖阳性（血糖正常）、氨基酸尿、低尿酸血症、低钙血症和低磷血症，以及高尿磷、代谢性酸中毒和骨软化。②血或尿中可检测到M蛋白，κ型轻链最为常见。③肾活检：光镜：肾小球病变轻微，肾小管上皮空泡样变性；免疫荧光：免疫荧光常阴性或单一轻链在近端小管内点状沉积；电镜：可见近端肾小管上皮细胞内晶状体沉积和溶酶体增多[16]。

预后和治疗：轻链近端肾小管病的肾脏病变进展缓慢。治疗方面，应积极纠正酸中毒和钙磷代谢异常。积极抗浆细胞治疗可逆转肾功能，改善临床症状[17]。

5. 硬化性黏液水肿：是一种慢性黏蛋白沉积伴纤维细胞增生及纤维化的临床综合征。临床表现主要为丘疹样皮疹和皮肤硬化，也可出现咽部和上气道受累、阻塞性肺病和肺动脉高压等[18]。

诊断：①广泛的丘疹样皮疹和皮肤硬化；②黏蛋白沉积伴成纤维细胞增生及纤维化；③IgG型M蛋白；④排除甲状腺疾病。

预后和治疗：以抗浆细胞治疗为主，局部治疗无效。

6. Clarkson病：又称特发性系统性毛细血管渗漏综合征。发病机制不明。

诊断：①有前驱期：多表现为发热、头痛、肌痛、乏力等；②有诱发因素；③发作期表现为分布性休克、高血红蛋白和低白蛋白血症；④恢复期可出现多尿及肺水肿；⑤自发缓解；⑥同时存在M蛋白，多为IgG-κ型，M蛋白在本病中作用机制不明；⑦除外其他继发因素[19]。

预后和治疗：主要死亡原因在于休克发作期没有得到及时救治。积极抗休克治疗可有效降低并发症发生率与死亡率。氨茶碱和特布他林可能有助于预防复发。抗浆细胞治疗可能有效。

7. TEMPI综合征

诊断：需要满足以下五联征：毛细血管扩张（telangiectasias），毛细血管扩张主要发生在面部、躯干和上肢；促红细胞生成素（EPO）升高和红细胞增多；M蛋白；肾周积液（perinephric fluid collections）；肺内分流（intrapulmonary shunting），肺内分流可造成低氧血症甚至呼吸衰竭[20]–[21]。部分患者还可以出现多浆膜腔积液。

预后和治疗：TEMPI综合征为慢性病程，预后相对较好，抗浆细胞治疗有效，少数难治复发患者也可考虑自体造血干细胞移植。

8. SLONM

诊断：①主要临床表现为进行性加重的肌无力、肌肉萎缩；②肌电图提示肌源性损害；③肌肉活检：光镜和电镜下可见杆状体；④成人起病，无家族史；⑤同时存在M蛋白，多为IgG型。

预后和治疗：发病时间短的患者早期治疗预后好，而发病时间较长患者预后较差。抗浆细胞治疗有效[22]。

9. 有肾脏意义的单克隆免疫球蛋白血症（MGRS）

MGRS指的是一组M蛋白直接或间接累及肾脏，并造成肾脏功能损害的疾病。诊断：①存在M蛋白；②存在肾脏病变；③M蛋白和肾脏病变之间存在着直接的因果关系。肾活检病理是诊断MGRS的关键，是建立M蛋白和肾病因果关系的主要手段；完整的肾活检病理至少应当包括光镜、免疫荧光（包括免疫球蛋白重链和轻链染色）和电镜[23]。

分类：根据电镜下的沉积物形态可将MGRS分为纤维素样沉积（如AL），微管样沉积（Ⅰ型和Ⅱ型冷球蛋白血症和免疫触须样肾病），无定形物质沉积（如轻链沉积病、重链沉积病、轻重链沉积病和伴免疫球蛋白沉积的增殖性肾病），晶状体样沉积（FS）及非沉积性疾病（POEMS综合征和C3肾炎）。

预后和治疗：治疗目标主要为保护并逆转肾功能，并预防移植肾复发。具体治疗方案可依据不同的致病细胞选择抗浆细胞治疗或去B细胞治疗。

10. CAD：为一种少见的冷抗体型自身免疫性溶血性贫血。

诊断：①有溶血性贫血的临床表现和体征；②Coombs试验：C3d阳性和（或）IgM阳性；③血中冷凝集素滴度≥1∶64；④除外其他感染和肿瘤导致的继发性CAD[24]。

预后和治疗：基于利妥昔单抗的联合化疗如苯达莫司汀联合利妥昔单抗，氟达拉滨、环磷酰胺联合利妥昔单抗或硼替佐米联合利妥昔单抗等。抗C1s单抗对于难治复发性CAD也有较好的疗效。BTK抑制剂对于难治复发性CAD也有效。糖皮质激素或脾切除的效果不佳[25]。

11. 冷球蛋白血症：冷球蛋白是一种当温度<37 °C时沉淀，当温度≥37 °C时可以重新溶解的免疫球蛋白。

诊断：①典型临床表现包括关节痛、皮肤紫癜、蛋白尿、血尿、肾功能不全以及周围神经病等；皮肤病理可见白细胞破碎性血管炎。肾脏病理可见膜增生性肾小球肾炎、毛细血管血栓形成或小血管炎表现。②血中存在冷球蛋白[26]。

分类：根据冷球蛋白的蛋白组成可分为Ⅰ型、Ⅱ型和Ⅲ型。Ⅰ型冷球蛋白血症为单克隆免疫球蛋白（IgG或IgM），常见于多发性骨髓瘤、巨球蛋白血症和淋巴瘤；Ⅱ型冷球蛋白血症为多克隆IgG和单克隆IgM，多见于丙型肝炎感染、自身免疫性疾病（如干燥综合征、系统性红斑狼疮、类风湿关节炎等）；Ⅲ型冷球蛋白血症为多克隆IgM和多克隆IgG。Ⅲ型冷球蛋白血症不属于MGCS范畴。

预后和治疗：治疗原发病为主。如丙型肝炎相关的冷球蛋白血症首先要抗丙肝治疗；MM相关需要抗骨髓瘤治疗。对于血管炎表现严重的可以予糖皮质激素、免疫抑制剂和（或）利妥昔单抗等免疫抑制治疗[27]–[28]。

12. IgM相关性周围神经病变（IgM-PN）

诊断：①周围神经病变：以缓慢进展的、对称性的、远端周围神经病为主要表现，感觉异常多见。少数也可表现为颅神经麻痹和单发或多发周围神经病。神经病理多为脱髓鞘病变。②IgM型M蛋白。③血抗髓磷脂相关糖蛋白（MAG）抗体阳性（约占50％）或抗神经节苷脂（GM）抗体阳性（占10％～20％）；抗体阴性不能排除IgM-PN可能。④除外冷球蛋白血症、肿瘤直接浸润或淀粉样变等引起的神经病变[28]。其中出现IgM-PN（GM抗体相关）、并发眼肌麻痹以及血冷凝集素阳性的患者则可诊断为CANOMAD综合征[29]。

预后和治疗：以含利妥昔单抗方案为主。对于发病时间短的患者，早期治疗可能逆转神经病变；对于发病时间较长患者，治疗并不能逆转神经病变。可以联合BTK抑制剂治疗[30]。

13. Schnitzler综合征：是一种罕见的MGCS。疾病发生可能与NLRP3基因突变造成IL-1β大量生成有关，M蛋白在疾病中的作用机制不明[31]。

诊断：强制性诊断标准：①反复发作的慢性荨麻疹；②IgM单克隆免疫球蛋白血症（主要为IgM-κ型，少见为IgG型）。次要标准：①反复发热；②提示骨骼异常重塑的客观指标（ALP升高或骨骼X线、MRI中看到成骨性改变）；③C反应蛋白水平升高；④白细胞增多；⑤皮肤活检可见白细胞浸润。诊断需满足2条强制性诊断标准和2条次要标准。若满足2条强制性诊断标准和1条次要标准，提示可能诊断。若为IgG型，则诊断需满足2条强制性诊断标准和3条次要诊断标准，满足2条强制性诊断标准和2条次要诊断标准提示可能诊断。

预后和治疗：首选白细胞介素受体拮抗剂（阿那白滞素）。15％～20％的患者最终可进展为淋巴增殖性疾病。
